# Understanding the mechanism of action and clinical effects of neuroactive steroids and GABAergic compounds in major depressive disorder

**DOI:** 10.1038/s41398-023-02514-2

**Published:** 2023-06-26

**Authors:** Andrew J. Cutler, Gregory W. Mattingly, Vladimir Maletic

**Affiliations:** 1 SUNY Upstate Medical University, Syracuse, NY USA; 2 Washington University, St. Louis, MO USA; 3 University of South Carolina, Columbia, SC USA

**Keywords:** Neuroscience, Molecular neuroscience

## Abstract

The pathophysiology of major depressive disorder (MDD) is thought to result from impaired connectivity between key brain networks. Gamma-aminobutyric acid (GABA) is the key inhibitory neurotransmitter in the brain, working primarily via GABA_A_ receptors, with an important role in virtually all physiologic functions in the brain. Some neuroactive steroids (NASs) are positive allosteric modulators (PAMs) of GABA_A_ receptors and potentiate phasic and tonic inhibitory responses via activation of synaptic and extrasynaptic GABA_A_ receptors, respectively. This review first discusses preclinical and clinical data that support the association of depression with diverse defects in the GABAergic system of neurotransmission. Decreased levels of GABA and NASs have been observed in adults with depression compared with healthy controls, while treatment with antidepressants normalized the altered levels of GABA and NASs. Second, as there has been intense interest in treatment approaches for depression that target dysregulated GABAergic neurotransmission, we discuss NASs approved or currently in clinical development for the treatment of depression. Brexanolone, an intravenous NAS and a GABA_A_ receptor PAM, is approved by the U.S. Food and Drug Administration for the treatment of postpartum depression (PPD) in patients 15 years and older. Other NASs include zuranolone, an investigational oral GABA_A_ receptor PAM, and PH10, which acts on nasal chemosensory receptors; clinical data to date have shown improvement in depressive symptoms with these investigational NASs in adults with MDD or PPD. Finally, the review discusses how NAS GABA_A_ receptor PAMs may potentially address the unmet need for novel and effective treatments with rapid and sustained antidepressant effects in patients with MDD.

## Introduction

Depression is a common and debilitating mental health disorder that negatively impacts a person’s health and functioning and is a leading cause of disability globally [1]. According to the Diagnostic and Statistical Manual of Mental Disorders, 5th edition, text revision, major depressive disorder (MDD) is characterized by ≥2 weeks of at least 5 of the following symptoms (at least 1 of which is depressed mood or anhedonia): depressed mood, anhedonia (loss of interest or pleasure in daily activities), feelings of guilt/low self-esteem, changes in sleep, weight loss or loss of appetite, psychomotor retardation or agitation, fatigue, poor concentration, and suicidal thought that represents a change from previous functioning. The major depressive episode must not be due to another disorder [2]. The global annual age-standardized prevalence of depressive disorders (MDD and dysthymia) in 2019 was estimated to be 3440.1 per 100,000 individuals [3]. Based on the 2021 National Survey on Drug Use and Health (NSDUH) in the United States, the annual prevalence of major depressive episodes in adults was 8.3% and that of major depressive episodes with severe impairment was 5.7% [4].

The etiology of depression has not yet been fully established, but considering the heterogeneity of symptoms, underlying genetics, and treatment responses, it is generally believed that the cause of MDD may be multifactorial. Various genetic and environmental factors (eg, first-degree family members with MDD, adverse childhood experiences, stressful life events) can influence the development of depression [2]. A genome-wide association study of genetic and health records of 1.2 million individuals from 4 separate data banks identified variations in 178 genes that were linked to MDD [5]. Stressful environmental signals can be integrated into the genome via epigenetic mechanisms, such as DNA methylation and histone modifications [6, 7]. Evidence also shows that modified DNA methylation patterns due to stress can affect brain plasticity and emotion in patients with depression [8]. Furthermore, brain imaging studies have shown structural and functional changes associated with depression [9–11]. Structural changes include a loss of glial cells, morphologic changes in neurons, and decreased volume in the cingulate cortex, prefrontal cortex (PFC), hippocampus, and amygdala [12]. Functional changes in MDD involve abnormal connectivity in the central executive, default mode, and salience networks, the key neuronal networks controlling mood, arousal, behavior, and cognition [13, 14]. Elevated activity of the hypothalamic-pituitary-adrenal (HPA) axis, neurotrophic deficit, and neuroinflammation are other potential mechanisms proposed for the development of depression [15, 16].

More recent data implicate alterations in the sensorimotor network as being the most consistent factor in depression [17, 18]. Evidence also suggests an association of altered connectivity in the default mode network with postpartum depression (PPD) [19]. These functional networks communicate using several neurotransmitters, including amino acids such as glutamate and gamma-aminobutyric acid (GABA), the primary excitatory and inhibitory neurotransmitters in the brain, respectively, and monoamines such as norepinephrine, dopamine, and serotonin [12, 20].

### Hypotheses of depression

Several hypotheses exist for the pathophysiology of depression as it relates to altered neurotransmitter levels. The early monoamine hypothesis, which posits that a core pathophysiologic feature of depression is depletion of brain monoamine neurotransmitters (eg, norepinephrine, dopamine, and serotonin), originated from the observation that most standard-of-care antidepressant therapies (ADTs) can increase extracellular concentrations of these neurotransmitters [15, 16, 21, 22].

The glutamatergic hypothesis of depression suggests an association between elevated glutamate levels and depression [16, 22]. This hypothesis is based on preclinical evidence of the antidepressant effects of N-methyl-D-aspartate (NMDA)-receptor antagonists [23]. Glutamate binds to NMDA receptors, resulting in excitatory neurotransmission [24]. Elevated glutamate levels lead to overactivation of NMDA receptors and induce calcium ion (Ca^2+^) influx, which in turn may lead to long-term potentiation and long-term depression [25, 26]. However, the evidence for elevated glutamate levels in depression is inconsistent. A postmortem study of adults with MDD reported increased glutamate levels in the frontal cortex of patients with MDD [27], and a proton magnetic resonance spectroscopy study showed increased glutamate levels in the occipital cortex of patients with MDD [28]. Conversely, a meta-analysis of proton magnetic resonance spectroscopy studies examining levels of glutamatergic neurometabolites reported significant decreases in the combined glutamine-plus-glutamate level within the medial PFC in patients with depression compared with healthy volunteers but not in the dorsolateral PFC or medial temporal cortex; differences in glutamate levels between the two groups were not significant in any of these areas [29]. Another meta-analysis showed that glutamate levels were lower within the anterior cingulate cortex of patients with depression compared with healthy volunteers [30].

The GABAergic deficit hypothesis proposes that defects in GABAergic neural inhibition causally contribute to the common phenotypes of MDD, and, conversely, the efficacy of an ADT may be linked to its ability to restore GABAergic neurotransmission [31]. It is based on findings of reduced levels of GABA in the plasma, cerebral cortex, and cerebrospinal fluid (CSF), altered expression and subunit composition of GABA_A_ receptors, and reduced levels of neuroactive steroids (NASs) in CSF among individuals with depression [22, 32]. The hypothesis is also supported by results from multidisciplinary contemporary approaches that combined large-scale genome-wide association studies, postmortem cytology, and functional and structural imaging studies to clarify the shared origins of otherwise biologically heterogeneous MDD [33, 34]. A consistent association was noted between principal neuroimaging findings in individuals with depression and downregulated genetic markers for cortical somatostatin-expressing GABAergic interneurons and astrocytes [33]. Polygenic somatostatin interneuron markers were most expressed in the subgenual anterior cingulate, medial PFC, anterior insula, and temporal lobes, coinciding with brain areas where imaging studies confirmed cortical thinning and aberrant connectivity in individuals with depression [33]. The expression of the MDD-associated somatostatin gene marker *SST* was found to be significantly negatively correlated with structural differences in cortical regions of individuals with MDD relative to healthy controls [34]. Impaired GABAergic signaling is also thought to be implicated in PPD [35, 36] and bipolar disorder [37, 38].

This narrative review provides preclinical and clinical data supporting the role of GABAergic signaling in the brain and the GABAergic deficit hypothesis of depression, and how modulation of GABA signaling by GABAergic compounds and NASs could potentially be employed to treat depression. We also examine currently approved and investigational NAS therapies and their hypothesized mechanisms of action in depression, supporting the potential link of science and practice for physicians and clinical researchers. The mechanisms and novel therapies reviewed may impact the approach to rapid treatment of MDD with improved long-term outcomes. Publications were selected from the literature based on their relevance to the covered topics (ie, the role of GABA signaling and the potential role of GABAergic compounds and NASs in MDD) and author experience and preference.

### GABAergic signaling and normal brain functioning

The complex interplay between excitatory glutamatergic neurons and inhibitory GABAergic neurons is essential to achieving balanced cortical neural activity [39, 40]. The glutamatergic-GABAergic balance is tightly regulated by the biosynthesis, transport, and signaling of the respective neurotransmitters (ie, glutamate and GABA) in the central nervous system (CNS) [39, 40]. The biosynthesis of glutamate and GABA are interrelated via the glutamate/GABA-glutamine cycle [41]. Briefly, glutamatergic neurons release glutamate via synaptic vesicles into the synaptic cleft, where it is taken up by astrocytes and converted to glutamine. Glutamine is transported back to glutamatergic neurons, hydrolyzed to glutamate, and repackaged into synaptic vesicles [41]. GABAergic neurons release GABA into the synapse, where it is taken up by astrocytes and ultimately converted to glutamine. Glutamine is then transported to GABAergic neurons, where it is converted to glutamate by glutaminase and then to GABA by glutamate decarboxylase [42].

### Physiologic role of GABA and GABAergic neurons

Excitatory glutamatergic and inhibitory GABAergic neurons predominantly communicate through synaptic interactions [43]. GABA is present primarily in local interneurons, but also in long projection neurons in the PFC, anterior cingulate cortex, amygdala, nucleus accumbens, ventral tegmental area, and the hippocampus—the regions functionally associated with decision-making, cognition, intelligence, memory, sleep, emotions, motivation, and pleasure [12, 44–47]. GABA plays an important role in neuronal proliferation, migration, differentiation, and preliminary circuit-building during brain development [44, 48] and is implicated in the development of interstitial neurons in white matter and oligodendrocytes [44]. GABA also regulates connectivity between the major brain functional networks (eg, default mode and executive control networks) [49].

**Fig. 1:**
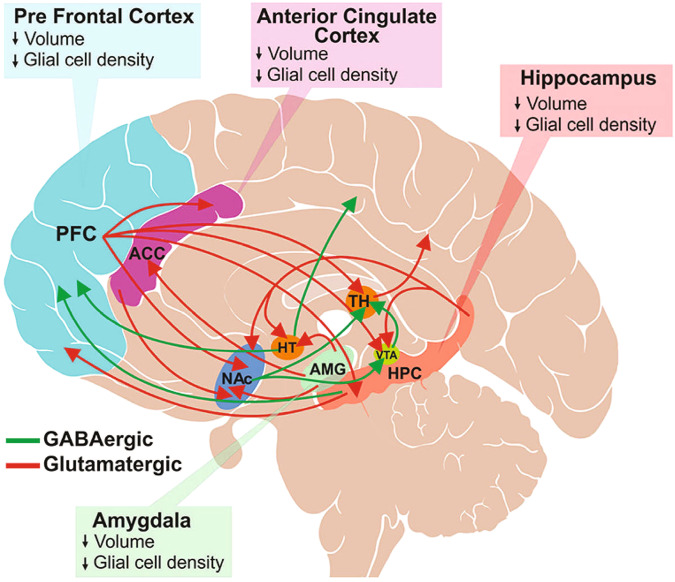
Glutamatergic and GABAergic projection neurons make dense connections between brain regions that participate in mood regulation and reward processing. Glutamatergic projections illustrated here include those from the frontal cortex to the anterior cingulate cortex (ACC), thalamus (TH), ventral tegmental area (VTA), hippocampus (HPC) and nucleus accumbens (NAc); from hippocampus to hypothalamus (HT), VTA, NAc and PFC; and from amygdala to HT, ACC and NAc. Major GABAergic projections are from HT to the occipital and parietal cortex, HPC to PFC, and from NAc to TH and VTA. Only a subset of known interconnections is shown here. Depression is associated with reduced brain volume and decreased glial cell density in various brain regions, including ACC, PFC, hippocampus, and amygdala. (Figure is reproduced from Sarawagi et al. 2021 [12] according to the terms of the Creative Commons Attribution License [CC BY]).

GABAergic projection neurons are widely distributed throughout the brain and make dense connections between brain regions involved in mood regulation and reward learning (Fig. 1) [12]; GABAergic interneurons play a vital role in local neural circuitry and activity [50]. Altogether, GABAergic neurons play an important role in regulating various physiologic brain functions, such as learning and memory, sensorimotor processing, and neuroplasticity [51]. GABAergic neurons also terminate the innate physiologic stress response by regulating the HPA axis and restoring homeostasis, suggesting the critical role of GABAergic signaling in normal brain function [45].

### GABA receptors

GABA mediates neural inhibition in the brain by activating the 2 major GABA receptors: (1) GABA_A_, ionotropic ligand-gated ion channels that signal via direct ligand-mediated opening; and (2) GABA_B_, metabotropic G protein-coupled receptors that act indirectly via intracellular signaling cascades [32, 52, 53]. GABA_B_ receptor-mediated signaling relies on the activation of G protein signaling pathways to inhibit neurotransmitter release and modulate action potential propagation [52, 54]. GABA_B_ receptor expression or activity does not appear to be consistently altered in individuals with depression and has therefore attracted less research interest compared with GABA_A_ receptors in this disease [55]. However, GABA_B_ receptor activation has been reported to increase membrane trafficking of GABA_A_ receptors in dentate gyrus granule cells, resulting in enhanced GABA_A_ receptor current [56, 57].

GABA_A_ receptors are widely distributed in the brain and play an important role in many brain functions [58, 59]. In addition to GABA, other endogenous ligands include zinc, NASs, and certain amino acids [60–62]. GABA_A_ receptors are encoded by 19 subunit genes, six α (α1-α6), three β (β1-β3), three γ (γ1-γ3), three ρ (ρ1-ρ3), and one each of the δ, ε, π, and θ subunits [63]. GABA_A_ receptors belong to a large heterogeneous class of pentameric chloride channels comprising 2 α, 2 β, and 1 γ, δ, ρ, θ, or ε subunits, with the α1β2γ2 GABA_A_ receptors being the most abundant [32, 53]. The complex and heterogenous nature of GABA_A_ receptors results in considerable diversity in their physiology, location, and pharmacologic profile [53]. Upon GABA binding and activation of the GABA_A_ receptor, chloride ions flow into the cell, leading to rapid membrane hyperpolarization and inhibition of action potentials in the postsynaptic neuron [52, 64]. A subclass of GABA_A_ receptors, termed GABA_A_-ρ (previously GABA_C_) receptors, is a group of receptors composed exclusively of ρ subunits, which are typically insensitive to GABA_A_ allosteric modulators (eg, benzodiazepines, barbiturates, and most NASs) [65]. However, there are some GABA_A_ receptor modulators that can also engage GABA_A_-ρ receptors, such as pregnanolone, allopregnanolone, and some synthetic NASs [65–67].

The subunit composition of the GABA_A_ receptor defines its biophysical and pharmacologic properties and whether it localizes to a synaptic or extrasynaptic site. The widely expressed α1–3β1–3γ2 GABA_A_ receptors are predominantly localized to the synapses, while the α4–6β2–3δ GABA_A_ receptors are largely present extrasynaptically [53, 68, 69]. Activation of low-affinity synaptic γ subunit-containing receptors is transient and mediates rapid phasic inhibition, while extrasynaptic δ subunit-containing receptors mediate tonic inhibition through persistent activation by low concentrations of ambient extracellular GABA [64, 70]. In neurons with both synaptic and extrasynaptic conductance, the tonic currents may produce a larger net inhibitory effect than do the phasic currents [71].

### Neuroactive steroids

**Fig. 2:**
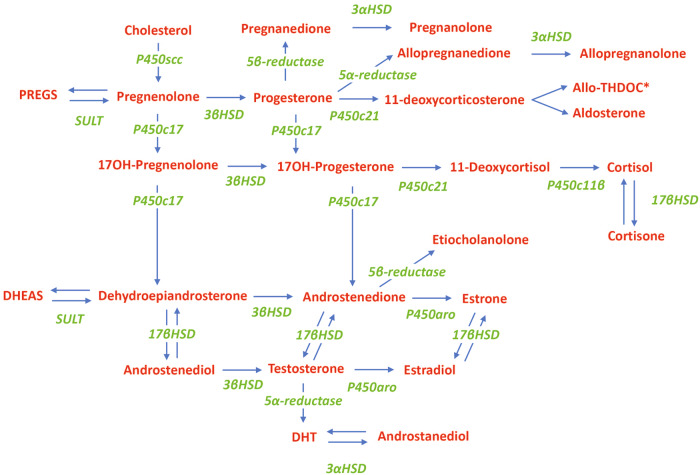
In the biosynthesis of NASs, cholesterol is translocated into the inner mitochondrial membrane by the steroidogenic acute regulatory protein. There it is metabolized by P450scc into pregnenolone, the precursor of all endogenous NASs. Biosynthetic enzymes are denoted in green; neuroactive steroids and substrates are denoted in red. *Allotetrahydrodeoxycorticosterone is also known as tetrahydrodeoxycorticosterone (same Chemical Abstract Services number). Allo-THDOC allotetrahydrodeoxycorticosterone, DHEAS dehydroepiandrosterone sulfate, DHT 5α-dihydrotestosterone, HSD hydroxysteroid dehydrogenase, NAS neuroactive steroid, P450aro cytochrome P450-aromatase, P450c11β cytochrome P450 11β-hydroxylase, P450c17 cytochrome P450 17α-hydroxylase, P450c21 cytochrome P450 21-hydroxylase, P450scc cytochrome P450 side chain cleavage, PREGS pregnenolone sulfate, SULT sulfotransferase.

Neuroactive steroids are a class of steroids that are synthesized de novo in neurons and glia of the central and peripheral nervous systems following transport of cholesterol into the mitochondria (Fig. 2). Additionally, some circulating sterols (eg, progesterone, dehydroepiandrosterone [DHEA]) can cross the blood-brain barrier to be used as precursor molecules [64, 72–75]. Endogenous NASs are generally categorized as: (1) pregnane-derived (eg, allopregnanolone, allotetrahydrodeoxycorticosterone [allo-THDOC]); (2) androstane-derived (eg, androstanediol, etiocholanolone); or (3) sulfated (eg, pregnenolone sulfate, dehydroepiandrosterone sulfate) [64, 72].

Neuroactive steroids regulate neuronal excitability via rapid non-genomic action [64], primarily through interaction with neuronal membrane receptors and ion channels like the ionotropic GABA_A_ receptors [64, 72]. Activity of NASs at neuronal GABA_A_ receptors occurs within minutes, compared with the slow-onset (delayed by hours) and prolonged duration of action of steroid hormones, which act via intracellular steroid hormone receptors [76]. In general, NASs bind to GABA, NMDA, serotonin, and σ-1 receptors to modulate neurotransmitter signaling [72]. They modulate excitatory-inhibitory balance and homeostatic mechanisms, thus regulating brain functions that control mood, aggression, cognition, memory, and pain [77]. Neuroactive steroids can function as positive allosteric modulators (PAMs) of both synaptic and extrasynaptic GABA_A_ receptors to activate and potentiate phasic and tonic currents, respectively [64, 78–80], or as negative allosteric modulators (NAMs) to dampen the response to neurotransmitter ligands such as glutamate and GABA [81]. NAS GABA_A_ receptor NAMs are activation-dependent, non-competitive inhibitors of GABA_A_ receptors and can also inhibit the effects of NAS GABA_A_ receptor PAMs [80]. NAS GABA_A_ receptor PAMs also regulate neuroplasticity, neuroinflammation, and HPA axis function and may play an important role in neurogenesis [36, 82–84]. Allopregnanolone, pregnanolone, and allo-THDOC are among the more potent endogenous NAS PAMs of GABAergic neurotransmission [85].

GABA_A_ receptor activation by NASs occurs via 2 discrete sites in the α and β subunit transmembrane domains, one at the α-β subunit interface for activation and the other exclusively on α subunits for potentiation of response to NASs [62, 79]. Binding of nanomolar concentrations of NAS GABA_A_ receptor PAMs increases the mean open time and decreases the mean closed time of the GABA_A_ receptor chloride channel in the presence of sub-saturating concentrations of GABA, thereby increasing the chloride current through the channel and reducing neuronal excitability [64, 86]. In the absence of GABA, micromolar concentrations of PAMs can directly open GABA_A_ receptor chloride channels [87]. Interestingly, NAS GABA_A_ receptor PAMs have also been shown to increase phosphorylation of certain GABA_A_ receptor subunits, leading to increased cell surface expression of those GABA_A_ receptors [88–90]

The sensitivity of GABA_A_ receptors to NASs is determined by the receptor subunit composition; at normal extracellular GABA concentrations, extrasynaptic δ subunit-containing GABA_A_ receptors are more sensitive to NAS modulation compared with synaptic γ subunit-containing GABA_A_ receptors [91], allowing for greater enhancement in GABA_A_ receptor currents [92]. This preferential interaction of NASs with extrasynaptic δ subunit-containing receptors is secondary to GABA acting as a partial agonist at these receptors [92]. However, allopregnanolone modulates both γ- and δ subunit-containing GABA_A_ receptors within a similar potency range and may therefore enhance both phasic and tonic currents, respectively [93].

### Dysregulated GABAergic signaling

Major depressive disorder has been linked to dysregulation of the excitatory-inhibitory balance within the brain and the reduced ability to maintain homeostasis in response to internal or external stimuli [12, 45, 94, 95]. Both preclinical and clinical data support an association of depression with diverse defects in GABAergic neurotransmission. Epigenetic changes of the GABAergic system have been shown to be responsible for adult hippocampus neurogenesis and depression-like behaviors in prenatal-stressed mice [96]. In addition, alterations in DNA methyltransferase mRNA expression have been observed in the brains of individuals with MDD who died by suicide compared with the brains of non-MDD/suicide individuals, and this change in expression was associated with gene-specific aberrations in DNA methylation in the GABA_A_ receptor α1 subunit promoter region within the frontopolar cortex [97]. Alterations in the DNA methylation signatures of GABA-related genes have also been reported in other psychiatric disorders, including autism spectrum disorder [98], schizophrenia [99, 100], psychosis with a history of chronic alcohol abuse [101], and bipolar disorder [102].

A review of postmortem studies found variable expression of various GABA_A_ receptor subunit mRNAs of suicide victims with depressive disorders and patients with MDD [32, 103]. A large human gene expression analysis of cortical and subcortical regions from the brains of depression-related suicides found that the expression levels of genes involved in GABAergic transmission were among the most consistently changed [104]. Genetic alteration of the γ-subunit of the GABA_A_ receptor disrupted the regulatory response of GABAergic neurons and led to depressive and anxiogenic behaviors in rodents [105–107]. Genetic studies in mice have shown that deletion of the GABA_A_ receptor γ subunit led to impaired GABAergic signaling and behavioral and cognitive deficits that could be reversed by chronic desipramine or acute ketamine [105, 108], while deletion of the α2 subunit led to depressive- or anxiety-like behaviors [109, 110]. Mice with decreased GABA_A_ receptor δ subunit expression displayed anxiety-like behavior and maternal neglect postpartum, and administration of the δ subunit selective agonist 4,5,6,7-tetrahydroisoxazolo[5,4-c]pyridin-3-ol (THIP) reduced the abnormal behaviors [111]. Similarly, administration of SGE-516, a NAS GABA_A_ receptor PAM, rescued these abnormal behaviors in this same model [112].

GABA levels and GABA_A_ receptor function were found to be diminished in several brain regions in rodent models of acute and chronic stress [113–116]. GABA_A_ receptor agonists or GABA_A_ receptor PAMs prevented and reversed rodent behavioral models of depression [117]; conversely, administration of GABA_A_ receptor antagonists to normal rodents caused behaviors that mimicked these models of depression [118]. Individuals with depression exhibit reduced functioning of GABAergic interneurons and defects in GABAergic neural inhibition compared with healthy controls [28, 119, 120]. Calbindin-D28K, a calcium-binding and buffering protein critical for preventing neuronal death as well as maintaining calcium homeostasis, is expressed ubiquitously across multiple brain regions that are intimately involved in regulating emotional behaviors, particularly in GABAergic interneurons in the PFC, amygdala, and hippocampus [121]. A postmortem study showed that the density and size of GABAergic interneurons immunoreactive for calbindin-D28K were significantly decreased in the PFC of individuals with MDD versus those without MDD [122]. Positron emission tomography imaging showed reduced GABA_A_ receptor binding of [^11^C]-flumazenil in the limbic parahippocampal temporal gyrus and right lateral superior temporal gyrus of individuals with MDD versus healthy controls, suggesting a decreased number of GABA_A_ receptors and/or reduced affinity to benzodiazepine-site ligands [123]. GABAergic inhibitory neurotransmission in cerebral cortex, as assessed using transcranial magnetic stimulation, has been shown to be reduced significantly in individuals with MDD [124].

Individuals with depression, compared with healthy controls, also exhibited diminished GABA levels in the brain, plasma, and CSF [38, 125, 126] that are most pronounced in melancholic and treatment-resistant depression [119, 127], and remission from MDD was accompanied by normalization of GABA levels in the brain [125]. Additionally, severity of anhedonia is inversely correlated with GABA levels in the anterior cingulate cortex as shown in adolescents with MDD [128, 129], further supporting the correlation between dysregulated GABAergic neurotransmission and depression. Findings of reduced levels of glutamate decarboxylase in postmortem PFC of individuals with untreated MDD compared with healthy controls provide additional evidence for a link between GABAergic dysfunction and depression [130]. ADTs that affect monoaminergic neurotransmission may also show downstream effects on GABA- and glutamatergic neurotransmission [22, 131]. In animal models, studies showed that selective serotonin reuptake inhibitors (SSRIs), serotonin and norepinephrine reuptake inhibitors (SNRIs), tricyclic antidepressants (TCAs), and monoamine oxidase inhibitors (MAOIs) decrease glutamatergic signaling [132–134]. Treatment with SSRIs or electroconvulsive therapy in individuals with depression has resulted in normalization of decreased plasma GABA levels in the brain and plasma [135–137]. Due to limited study group sizes, no significant correlation was found between measures of clinical response and the change in cortical GABA concentrations in 2 of these studies [136, 137]. However, in a study of inpatients with MDD treated with SSRIs, 70% of responders had increased GABA levels and 64% had decreased glutamate levels [135].

Dysregulated GABA neurotransmission is also linked to anxiety and insomnia, 2 common comorbidities in individuals with depression. An association between anxiety and GABAergic signaling is supported by the preclinical findings of anxiety behaviors related to chronic inhibition of GABA synthesis [138] and the disruption of the anxiolytic-like effect of diazepam due to diminished levels of glutamate decarboxylase [139]. Additionally, review of the therapeutic mechanism of action for different anxiolytics found that these drugs may share a final common pathway involving enhancement of GABAergic neurotransmission [131]. GABA is also believed to be involved in the regulation of sleep [140]. Time awake after sleep onset has been found to be inversely correlated with GABA levels in individuals with primary insomnia, although results regarding changes in GABA levels of individuals with primary insomnia versus healthy controls are inconsistent [141, 142]. Furthermore, drugs targeting GABA_A_ receptors, such as benzodiazepines and *Z*-drugs, exhibit sedative and hypnotic effects [143, 144].

**Fig. 3:**
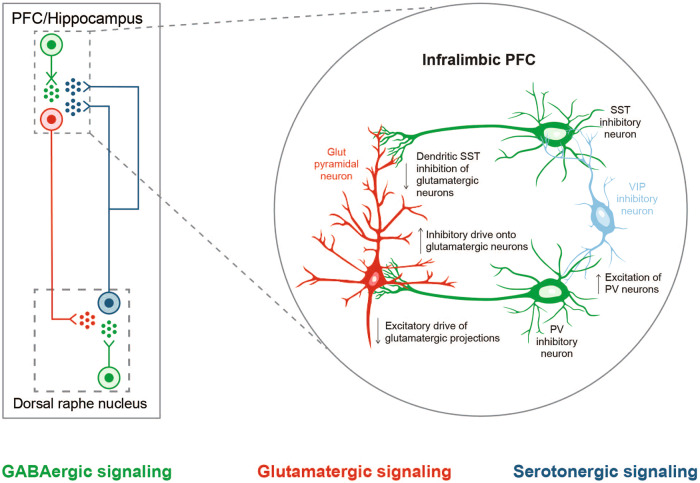
The GABAergic and the monoaminergic neurons are interconnected. Serotonergic neurons originating in the dorsal raphe nucleus and projecting to the prefrontal cortex (PFC) regulate excitability of GABAergic and glutamatergic neurons, which in turn, modulate the excitability of serotonergic neurons in the dorsal raphe nucleus by the GABA-glutamate balance (left). Chronic stress affects local networks regulating activity within the medial PFC (mPFC), leading to changes in local excitatory-inhibitory balance. In a proposed mechanistic model (right), somatostatin-expressing GABAergic neurons provide reduced dendritic inhibition of glutamatergic pyramidal neurons in the infralimbic mPFC under chronic stress, reducing filtering of information flow into the PFC [145]. An altered glutamate and GABA neurotransmission might appear as a disturbance in monoamine signaling. (Part of this figure is adapted from McKlveen et al. 2019 [145], with permission from Elsevier).

GABAergic and monoaminergic neurons are interconnected, and, consequently, GABA_A_ receptor deficits can also alter dopaminergic, serotonergic, and noradrenergic activity [32] (Fig. 3). Additionally, inadequate signaling in somatostatin-positive GABAergic interneurons in prefrontal microcircuits (established as one of the key substrates in MDD) can potentially produce attenuated pyramidal neuron output from the PFC and subsequent downstream regulation of threat and danger circuits (amygdala and bed nucleus of the stria terminalis) and sensory and motor processing in the thalamus, mimicking monoamine insufficiency in the brainstem [145, 146].

### Role of neuroactive steroids in depression and other brain disorders

The downregulated biosynthesis of NAS GABA_A_ receptor PAMs has been implicated in various psychiatric disorders (eg, MDD, PPD, premenstrual dysphoric disorder, and posttraumatic stress disorder [PTSD]) [31, 147]. Changes in NAS GABA_A_ receptor PAMs synthesis pathways have been linked to the pathologies of neurodegenerative and inflammatory brain diseases (eg, Alzheimer’s disease, Parkinson’s disease, multiple sclerosis) based on postmortem studies [148], as well as to self-reported pain symptoms (eg, chest pain, muscle soreness) [149] in humans. Levels of allopregnanolone have been shown to be significantly decreased in individuals with PTSD, a condition that is highly comorbid with MDD [150].

Reduced levels of allopregnanolone in the CSF and plasma have also been reported in individuals with mood disorders such as MDD, in addition to decreased GABA levels [151, 152] and decreased PFC expression of 5α-reductase, the enzyme catalyzing the rate-limiting step in allopregnanolone biosynthesis [153]. Decreased levels of allopregnanolone in the plasma or serum were also found in individuals with postpartum “blues” or pharmacologically induced panic attacks [154, 155], in contrast to the increased level of the 3β isomer of allopregnanolone, which antagonizes GABA_A_ receptor function in panic attacks [148]. Significant fluctuations in the blood and brain levels of allopregnanolone were shown to be strongly correlated with alterations in function and plasticity of GABA_A_ receptors in rodents [156, 157]. The failure to upregulate GABA_A_ receptors in response to the rapid drop in levels of allopregnanolone postpartum is likely involved in the development of PPD [35].

While acute stressors can lead to increased levels of NAS GABA_A_ receptor PAMs (ie, allopreg-nanolone and allo-THDOC) in animal models [36, 158], chronic stress, a major predictor of MDD, was shown to result in altered GABAergic signaling and decreased production of endogenous GABA_A_ receptor PAMs (ie, allopregnanolone) in rodent stress models [80, 159, 160]. Chronic stress-induced reduction in allopregnanolone levels was associated with abnormal behaviors such as aggression, enhanced fear, depressive- or anxiety-like behaviors, and impaired adult hippocampal neurogenesis in animal models [161–164].

Selective serotonin reuptake inhibitors such as fluoxetine and norfluoxetine can normalize decreased levels of allopregnanolone in the brain while decreasing behavioral abnormalities associated with mood disorders, as demonstrated in socially isolated mice [165, 166]. Studies in individuals with depression also showed that treatment with fluoxetine could increase allopregnanolone levels in the CSF [151, 152], and these changes were correlated with improvements in depressive symptoms [151]. The important role of NAS GABA_A_ receptor PAMs in depression is further supported by the findings that allopregnanolone administration prevented or normalized depressive- or anxiety-like behaviors in a social isolation rodent model [163].

### GABA_A_ receptor positive allosteric modulators

**Fig. 4:**
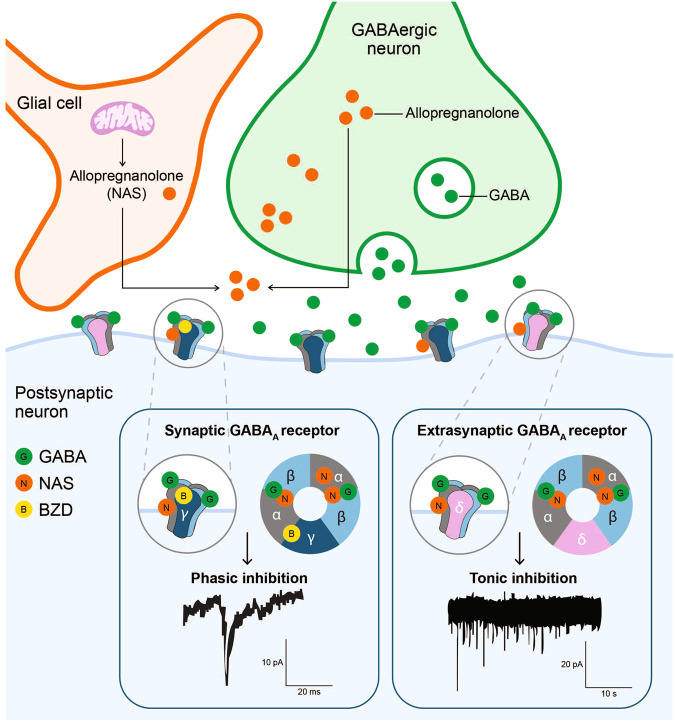
Neuroactive steroid (NAS) positive allosteric modulators (PAMs) of gamma-aminobutyric acid type A (GABA_A_) receptors amplify the inhibitory signal of gamma-aminobutyric acid (GABA) in the brain. NAS GABA_A_ receptor PAMs, such as allopregnanolone, bind to GABA_A_ receptors at sites distinctive from those for benzodiazepines (BZDs). NAS GABA_A_ receptor PAMs bind to both synaptic γ subunit-containing and extrasynaptic δ subunit-containing GABA_A_ receptors, potentiating phasic and tonic currents, respectively. In contrast, benzodiazepines bind to γ subunit-containing GABA_A_ receptors only and primarily augment phasic inhibition. Extrasynaptic GABA_A_ receptors containing δ subunits are insensitive to benzodiazepines [32, 53, 62, 64, 78, 79, 91, 92, 167].

Most GABA_A_ receptor-targeting drugs (ie, barbiturates, benzodiazepines, and NASs) function via allosteric binding to the receptor at sites distinct from the GABA binding sites (Fig. 4) [58, 62, 79, 91, 167, 168]. GABA binding sites are located at the α-β subunit interface on both synaptic and extrasynaptic receptors [58]. Barbiturates, benzodiazepines, and NASs bind the GABA_A_ receptor at allosteric sites and increase the GABA_A_ receptor current by increasing chloride conductance [169]. The presence of GABA is necessary for benzodiazepine response. Binding of benzodiazepines to the synaptic GABA_A_ receptor locks the receptor into a conformation for which GABA has much higher affinity, thus increasing the frequency of the chloride channel opening, with minimal effect on the duration of bursts [170, 171]. Barbiturates, on the other hand, bind in the presence of GABA to both synaptic and extrasynaptic GABA_A_ receptors and increase the duration of chloride channel opening without altering the frequency of bursts [169, 171]. Only at high doses can barbiturates directly stimulate GABA_A_ receptors in the absence of GABA [172].

Barbiturates bind to the α-β subunit interface on synaptic and extrasynaptic receptors and to γ-β subunit interfaces on synaptic receptors [58]. Barbiturates also non-selectively bind to the entire superfamily of ligand-gated ion channels [173]. Barbiturates act as antagonists of ionotropic glutamate receptors, such as α-amino-3-hydroxy-5-methyl-4-isoxazolepropionic acid (AMPA) and kainate receptors, thus inhibiting the bulk of fast excitatory synaptic transmission and glutamate release throughout the CNS [173]. These actions may account for the antidepressant, anxiolytic, hypnotic, and anticonvulsant activities of barbiturates, but may also account for the physical and psychological addiction potential and high rates of tolerance and dependence associated with this class of drugs. Barbiturates also have a high overdose potential due to a very narrow dosage margin [174]. Barbiturates were used to treat depression, anxiety, and insomnia in the early part of the 20th century but were generally replaced by benzodiazepines in the 1960s because the many potential drawbacks outweighed their usefulness [175].

Benzodiazepines are sometimes used to treat specific symptoms that are frequently associated with depression (eg, anxiety and insomnia). However, while a meta-analysis showed that treatment with the benzodiazepine alprazolam led to a higher percentage of individuals with MDD achieving response on the 17-item Hamilton Rating Scale for Depression (HAMD-17; ≥50% reduction in total score) or Clinical Global Impression-Improvement (CGI-I) scale (much improved or very much improved) compared with placebo, other benzodiazepines such as chlordiazepoxide and diazepam did not exhibit clear antidepressant activity [176]. In addition, chronic use of benzodiazepines more than 2–4 weeks is not recommended as it may result in decreased GABAergic and monoaminergic function, cognitive and psychomotor impairment, and interference with neurogenesis [177, 178]. These concerns, in addition to the risk of dependence and abuse [179] and an overall increase in the risk of attempting or completing suicide [180] may further limit the potential use of benzodiazepines in the treatment of depression [178].

Given that benzodiazepines appear to have questionable antidepressant activity, it is speculated that lack of antidepressant activity may be associated with the subunit composition of the GABA_A_ receptors with which benzodiazepines interact. Benzodiazepines bind at the α-γ subunit interface on synaptic GABA_A_ receptors (primarily augmenting phasic inhibition) [58]. Preclinical data suggest that α1 subunit-containing GABA_A_ receptors play a major role in sedation and addiction [181, 182], positive modulation of α2 subunit-containing receptors may have more consistent antidepressant effects [109], and activation of α3 subunit-containing receptors have pro-depressant actions [183]. Benzodiazepines show a higher affinity for α1 subunit-containing receptors [53] and concomitantly activate α2- or α3 subunit-containing receptors [184], the net effect of which is the promotion of sedation and addiction and a null effect on depression. In addition, benzodiazepines may only enhance phasic inhibitory currents through their binding to synaptic (γ subunit-containing) GABA_A_ receptors [185]. GABA_A_ receptor PAMs that bind to both synaptic and extrasynaptic GABA_A_ receptors and enhance both phasic and tonic inhibitory currents, respectively, may have greater therapeutic utility than benzodiazepines in treating depression.

The ability of NASs to target both synaptic and extrasynaptic GABA_A_ receptors is especially important in conditions where synaptic GABA_A_ receptors are downregulated, a condition which could lead to benzodiazepine tolerance [185]. NASs can interact with most GABA_A_ receptors, including the benzodiazepine-insensitive receptors containing α4 and α6 subunits or lacking the γ subunit [91]. In addition to allosteric modulation of GABA_A_ receptors, NAS GABA_A_ receptor PAMs can also exert metabotropic effects on GABAergic inhibition via activation of the G protein-coupled membrane progesterone receptors (mPRs); mPR-dependent modulation of GABA_A_ receptor phosphorylation results in increased cell surface expression of GABA_A_ receptors and thus a sustained elevation in tonic current [90], further differentiating them from benzodiazepines, which are associated with a downregulation of GABA_A_ receptors [186]. The tonic current is resistant to the competitive GABA_A_ receptor antagonist gabazine, confirming that it is not generated from GABA binding to these receptors [187].

## Current treatment options for mdd in clinical practice

The 8 general groups of approved drugs for MDD are: SSRIs, SNRIs, TCAs, tetracyclic antidepressants (TeCAs), MAOIs, atypical and multimodal antidepressants, NMDA receptor antagonists, and GABA_A_ receptor modulators (Table 1) [188–194].

**Table 1:** Antidepressants in Current Clinical Practice and Their Hypothetical Mechanisms of Action in Depression.

Class	Mechanism of action	Common side effects
SSRI	Selectively inhibit serotonin transporters at the presynaptic neuronal axon terminal, thereby limiting serotonin reuptake [195]	Sexual dysfunction, sleep disturbances, weight changes, anxiety, dizziness, dry mouth, headache, fatigue, gastrointestinal distress [188, 195]
SNRI	Prevent the reuptake of both serotonin and norepinephrine by blocking both of their presynaptic transporters [196]	Nausea, insomnia, dry mouth, headache, increased blood pressure, sexual dysfunction, weight gain, dizziness, fatigue, somnolence, constipation, sweating [188, 238]
TCA and TeCA	Block the reuptake of serotonin and norepinephrine at the presynaptic neuronal terminals [191, 239]	Weight gain, sedation, dry mouth, nausea, blurred vision, constipation, confusion, urinary retention, tachycardia, dizziness, increased appetite [188, 239]
MAOI	Block monoamine oxidase enzyme that breaks down monoamine neurotransmitters (eg, norepinephrine, serotonin, dopamine, histamine) [240, 241]	Dry mouth, nausea, diarrhea, constipation, drowsiness, insomnia, dizziness, weight gain, fatigue, sexual dysfunction, hypotension [188, 240]
Serotonin modulator	Inhibit the presynaptic reuptake of serotonin and modulate various 5-HT receptor subtypes [192–194]	Vary by agent but commonly include sexual dysfunction, diarrhea, nausea, dizziness, headache, sedation [192–194]
Atypical and Multimodal	Various mechanisms of action (eg, NDRI; block α2 adrenergic receptors/antagonize the 5-HT receptor; antagonize serotonergic 5-HT2C receptors; inhibit monoamine reuptake/antagonize nicotinic Ach receptor; antagonize NMDA receptor/activate σ-1 receptor) [190, 192–194, 242]	Vary by agent, but commonly include dry mouth, dizziness, lightheadedness, headache blurred vision, sedation, constipation, low blood pressure, confusion, weight gain, nausea [191–194, 243]
NMDA receptor antagonist (ketamine/ esketamine)	Bind to and inhibit NMDA receptors on GABAergic interneurons, which leads to a transient surge in glutamate release and AMPA receptor upregulation, triggering the release of brain-derived neurotrophic factor and a wave of synaptogenesis [190, 205]	Dissociation, dizziness, sedation, nausea/vomiting, vertigo, anxiety, hypoesthesia, lethargy, blood pressure increased, feeling drunk, euphoric mood, blurred vision, headache, dry mouth, restlessness [207, 244]
GABA_A_ receptor modulator (brexanolone^a^)	Augment GABA_A_ receptor activity through positive allosteric modulation of the receptors [31]	Sedation/somnolence, dry mouth, loss of consciousness, flushing/hot flush [191, 211]

^a^Approved specifically for postpartum depression.

*5-HT* 5-hydroxytryptamine (serotonin), *Ach* acetylcholine, *GABA*_*A*_ gamma-aminobutyric acid type A, *MAOI* monoamine oxidase inhibitor, *NDRI* norepinephrine and dopamine reuptake inhibitor, *NMDA* N-methyl-D-aspartate, *SNRI* serotonin and norepinephrine reuptake inhibitor, *SSRI* selective serotonin reuptake inhibitor, *TCA* tricyclic antidepressant, *TeCA* tetracyclic antidepressant.

Standard-of-care ADTs used in the current pharmacologic management of MDD primarily target monoamine neurotransmitter systems. Compared with first-generation ADTs (TCAs and MAOIs), SSRIs and SNRIs have generally been shown to cause relatively fewer adverse effects and therefore appear to be more widely used [195, 196]. A meta-analysis of the efficacy and tolerability of SSRIs against TCAs in patients with MDD showed that although TCAs demonstrated similar efficacy with SSRIs (with superior efficacy in hospitalized patients), they are associated with significantly more adverse effects due to their inhibition of cholinergic, α-1 adrenergic, and histaminergic receptors [197]. TCAs are also more likely to induce toxicity and can be fatal if overdosed [198]. Although efficacious, MAOIs are not commonly prescribed because of potentially fatal reactions including increased blood pressure, heart attack, stroke, or serotonin syndrome when used together with foods containing high levels of tyramine (eg, aged cheese, spoiled meat, soy sauce) or other drugs [199, 200]. Monoaminergic ADTs often require 4 to 6 weeks or longer to take effect [201–203]. In addition, the STAR*D Study has shown that as many as approximately 50% of patients may not respond adequately [203]. Relapse rates can be high in patients taking standard-of-care ADTs, especially in those who require multiple treatment steps, as demonstrated in the STAR*D Study among patients with MDD (relapse rates ranged from 40%–70% during a 12-month naturalistic follow-up) [202].

Novel ADTs with targets that have been implicated in the neurobiology of depression beyond monoamines (eg, glutamate and GABA), are being investigated. For example, while the mechanism of action for the antidepressant effects of ketamine is not fully understood, it is thought that it may block NMDA receptors on GABAergic interneurons, thereby preventing their activation [204]. Subsequently, downstream disinhibition of glutamatergic neurons causes a glutamate surge. Increased extracellular glutamate initiates activation of postsynaptic AMPA receptors, leading to potentiation of BDNF and mTORC1 synaptogenic signaling pathways (Table 1) [190, 204, 205]. In addition, one study found that the antidepressant effects of ketamine were blocked when naltrexone, an opioid antagonist, was administered prior to ketamine [206], suggesting that the antidepressant effect of ketamine may be dependent on opioid receptor activation and not necessarily due to neurological actions mediated by NMDA receptors. Ketamine’s S-enantiomer, esketamine, was recently approved by the U.S. Food and Drug Administration (FDA) for treatment-resistant depression and MDD with acute suicidal ideation or behavior [207]. In contrast to standard monoaminergic ADTs, ketamine has demonstrated rapid antidepressant effects that peak at approximately 24 h and are sustained for approximately 1 week after administration in adults with MDD or bipolar depression [208]. However, the long-term use of ketamine may induce urologic toxicity [209], and chronic abuse of ketamine can negatively affect brain structure and functioning and cause cognitive impairment [210].

Newer ADTs targeting dysregulated GABA neurotransmission are also being developed. These include GABA_A_ and GABA_B_ receptor modulators (allosteric modulators, NASs, agonists, and antagonists), and GABAergic interneuron-targeting neuropeptides [45].

### Neuroactive steroids for treatment of depression

Among the more recent additions to the treatment landscape, brexanolone, a NAS GABA_A_ receptor PAM that is chemically identical to endogenous allopregnanolone (Table 1), was approved in 2019 by the FDA to treat adults with PPD (Table 2) [211]. This indication was expanded in 2022 to include patients ≥15 years of age [212]. Prior to the approval of brexanolone, the standard of care for PPD was psychotherapy, psychotropics, or combination treatment. Medications adapted from MDD treatment but not specifically approved for PPD included SSRIs, SNRIs, and TCAs [213]. In pivotal phase 2 and 3 clinical trials, adult women with PPD who received brexanolone demonstrated significant improvement in depressive symptoms compared with those who received placebo; improvement was rapid (at Hour 60) and sustained (through day 30) [214–216]. Brexanolone was generally well tolerated in these trials [214, 216]. An intravenous preparation of brexanolone was used because of low oral bioavailability and high in vivo clearance of endogenous allopregnanolone [216]. While the use of brexanolone can be limited by the relatively long, continuous infusion time (60 h), these data have led to an increased interest in the therapeutic potential of GABA_A_ receptor-modulating NASs.

**Table 2:** Neuroactive Steroid-based Drugs for the Treatment of Depression That Have Been Approved or are Currently Under Investigation.

Drug	Structure	Development phase	Indication
Brexanolone		FDA-approved in 2019 [211]	PPD
Zuranolone		Phase 3	PPD MDD
PRAX-114	Not available	Phase 2/3^a^	MDD
PH10		Phase 2a	MDD

^a^The phase 2/3 trial was completed and failed to meet its primary endpoint.

*FDA* US Food and Drug Administration, *MDD* major depressive disorder, *PPD* postpartum depression.

Another NAS, PRAX-114 is a primarily extrasynaptic GABA_A_ receptor PAM in oral formulation that was being investigated for the treatment of MDD [217, 218] (Table 2). Interim results from a non–placebo-controlled, 3-arm, fixed-dose, phase 2 safety and tolerability study conducted in Australia showed improvements from baseline in depression severity as assessed by HAMD-17 total score reductions following a 14-day treatment course with PRAX-114 [219]. Changes from baseline (CFB) in the HAMD-17 total score (reductions of 15–19 points) were observed in all 3 arms over an 8-day period. The safety and efficacy of a 28-day treatment course with PRAX-114 as monotherapy for severe MDD were also assessed in the phase 2/3, randomized, double-blind, placebo-controlled Aria trial (*N* = 216) [217]. However, this study failed to meet its primary endpoint of CFB in HAMD-17 total score on day 15, nor did it meet any of the secondary endpoints [220]. The sponsor has closed screening in its randomized, double-blind, placebo-controlled phase 2 trial as adjunctive and monotherapy treatment for patients with MDD and inadequate response to antidepressant treatment (*N* = 110), has stopped enrollment in a PTSD phase 2 trial, and has discontinued an essential tremor trial. The sponsor has no plans to pursue further development of PRAX-114 for psychiatric disorders.

PH10 is an investigational, synthetic NAS from the family of pherines, formulated as a nasal spray, currently under clinical development for treatment of MDD [221] (Table 2). PH10 acts on nasal chemosensory receptors to modulate neural circuits in the brain, including connections to the limbic amygdala and other basal forebrain structures, leading to antidepressant effects [221]. In a 3-arm (high-dose, low-dose, and placebo) phase 2a pilot study in patients with MDD (*N* = 30), treatment with PH10 led to a greater improvement in depressive symptoms as assessed by CFB (reductions) in HAMD-17 total score compared with placebo after 8 weeks of treatment, with minimal side effects and potentially a rapid (week 1) onset of effects [221, 222]. Mean CFB in HAMD-17 total score at week 8 were 17.8 (high dose), 16.3 (low dose), and 10.9 for placebo (overall *p* = 0.07; high dose *p* = 0.02; low dose *p* = 0.10). HAMD-17 responder rates of the 3 doses at week 8 were 80% (high dose; *p* > 0.05), 90% (low dose; *p* > 0.05), and 60% (placebo), and remission rates were 60% (*p* > 0.05), 80% (*p* < 0.05), and 20%, respectively. Adverse events that were more common with PH10 compared with placebo included increased appetite, daytime sleepiness, nasal dryness, headache, and bitter taste. A phase 2b trial of PH10 nasal spray for the treatment of MDD has been planned [223]. In addition, future development as a treatment for PPD, treatment-resistant depression, and suicidal ideation is under consideration [224].

Zuranolone is an oral, investigational, synthetic NAS and PAM of both synaptic and extrasynaptic GABA_A_ receptors that upregulates GABA_A_ receptor expression and enhances inhibitory GABAergic signaling [225]. It is currently in clinical development and being investigated as an oral, 14-day treatment for adults with MDD or PPD (Table 2). Zuranolone has a pharmacokinetic profile that enables oral once-daily dosing with increased bioavailability [226, 227]. In two phase 3 trials in adults with PPD assessing zuranolone 30 mg (*N* = 150) or zuranolone 50 mg (*N* = 195), those who received a once-daily, 14-day treatment course of zuranolone demonstrated significant improvements in depressive symptoms as assessed by CFB (reductions) in HAMD-17 total score at day 15 compared with those who received placebo [228, 229]. Mean CFB in HAMD-17 total score at day 15 were 17.8 (vs placebo 13.6; *p* < 0.05) with zuranolone 30 mg and 15.6 (vs placebo −11.6; *p* < 0.05) with zuranolone 50 mg. Rapid (day 3) and sustained (day 45) improvements in depressive symptoms were significantly greater with zuranolone than with placebo (*p* < 0.05) in both studies. HAMD-17 response rates at day 45 (end of study) were 75.3% (vs placebo 56.5%; nominal *p* > 0.05) and 61.9% (vs placebo 54.1%; nominal *p* > 0.05), and remission rates were 53.4% (vs placebo 30.4%; nominal *p* < 0.01) and 44.0% (vs placebo 29.4%; nominal *p* > 0.05) in the 2 studies [228, 229]. In a phase 2 trial (zuranolone 30 mg, *N* = 89) and a phase 3 trial (zuranolone 50 mg, N = 534) in adults with MDD, those who received treatment with zuranolone demonstrated significantly greater improvements in depressive symptoms as assessed by CFB (reductions) in HAMD-17 total score at day 15 compared with those who received placebo [230, 231]; mean CFB in HAMD-17 total score at day 15 were 17.4 (vs placebo 10.3; *p* < 0.05) and 14.1 (vs placebo −12.3; *p* < 0.05), respectively. Another phase 3 trial assessing zuranolone 20 mg (*N* = 194) and 30 mg (*N* = 194) in patients with MDD did not meet its primary endpoint [232]; mean CFB in HAMD-17 total score at day 15 was 12.5 with zuranolone 30 mg compared with 11.1 with placebo (*N* = 193) (*p* > 0.05). Rapid (by day 2 or 3) improvement in depressive symptoms were observed in these 3 trials in MDD (nominal *p* < 0.05 vs placebo) [233]. HAMD-17 response rates at day 42 (end of study) were 61.9% (vs placebo 56.4%; nominal *p* > 0.05) in the phase 2 trial, 52.9% (vs placebo 45.9%; nominal *p* > 0.05) in the phase 3 zuranolone 50 mg trial, and 43.4% (vs placebo 41.5%; nominal *p* > 0.05) in the phase 3 zuranolone 20 or 30 mg trial; HAMD-17 remission rates were 45.2% (vs placebo 33.3%; nominal *p* > 0.05), 30.8% (vs placebo 29.6%; nominal *p* > 0.05), and 24.3% (vs placebo 25.9%; nominal *p* > 0.05), respectively [230–232]. Use of standard ADTs at baseline was allowed in these trials, providing the patient was on a stable dose. Zuranolone as a co-initiation therapy was evaluated in a phase 3 trial; rapid and significantly greater improvement from baseline in HAMD-17 total score was observed at day 3 with zuranolone versus placebo when co-initiated with standard-of-care ADTs in adults with MDD (*p* < 0.001) [234]. Moreover, in an ongoing open-label study that includes assessment of the need for repeat treatment courses with zuranolone over 1 year, of enrolled adults with MDD who responded at day 15 to treatment (≥50% reduction from baseline in HAMD-17 score) with an initial 14-day treatment course of zuranolone 50 mg and continued beyond day 28, 79.5% received a total of 1 or 2 treatment courses during their time of up to 1 year in the study [235, 236]. Zuranolone was generally well tolerated. In clinical trials, adverse events that were more common (>5% in zuranolone) with zuranolone compared with placebo included somnolence, dizziness, sedation, and fatigue. The overall incidence of serious adverse events was low, reported in <2% of zuranolone-treated patients across the trials. No patient enrolled in any clinical trial to date (February 2023) has reported developing withdrawal syndrome after discontinuation of zuranolone.

## Conclusions

Treatment responses to standard-of-care oral antidepressants have been suboptimal in many individuals with MDD, potentially due to slow onset of effects, low response rates, adverse effects, and the need for chronic treatment. There remains an unmet need for novel and effective treatments with rapid, robust, and sustained antidepressant effects; with better safety and tolerability than standard-of-care ADTs, and ideally without the need for chronic treatment. The development of novel therapeutics for MDD relies on a deep, comprehensive, and evolving understanding of the pathophysiology of depression.

There has been increased interest in GABA_A_ receptor-based treatment approaches for MDD. Recent research on the proposed mechanism of action of NASs for PPD and MDD underscores the potential role of GABAergic signaling in the pathophysiology of depression. Although the placebo effect in depression may be a factor associated with failure to establish efficacy of novel treatments in clinical trials [237], data reviewed here indicate that NAS GABA_A_ receptor PAMs may potentially offer rapid and sustained antidepressant benefits for individuals with MDD. Further research is necessary to better understand the role of NAS GABA_A_ receptor PAMs in MDD.
